# Impact of COVID-19 on PM_2.5_ Pollution in Fastest-Growing Megacity Dhaka, Bangladesh

**DOI:** 10.1017/dmp.2021.131

**Published:** 2021-04-30

**Authors:** Showmitra Kumar Sarkar, Md Mehedi Hasan Khan

**Affiliations:** 1 Department of Urban and Regional Planning, Khulna University of Engineering & Technology (KUET), Khulna, Bangladesh; 2 REACH Initiative, Cox’s Bazar, Bangladesh

**Keywords:** novel coronavirus, pandemic, particulate matter, air pollution, South-Asia

## Abstract

**Objective:**

The purpose of the research was to investigate and identify the impact of COVID-19 lockdown on fine particulate matter (PM_2.5_) pollution in Dhaka, Bangladesh by using ground-based observation data.

**Methods:**

The research assessed air quality during the COVID-19 pandemic for PM_2.5_ from January 1, 2017 to August 1, 2020. The research considered pollution in pre-COVID-19 (January 1 to March 23), during COVID-19 (March 24 to May 30), and post-COVID-19 (May 31 to August 1) lockdown periods with current (2020) and historical (2017-2019) data.

**Results:**

PM_2.5_ pollution followed a similar yearly trend in year 2017-2020. The average concentration for PM_2.5_ was found 87.47 μg/m^3^ in the study period. Significant PM_2.5_ declines were observed in the current COVID-19 lockdown period compared with historical data: 11.31% reduction with an absolute decrease of 7.15 μg/m^3^.

**Conclusions:**

The findings of the research provide an overview of how the COVID-19 pandemic affects air pollution. The results will provide initial evidence regarding human behavioral changes and emission controls. This research will also suggest avenues for further study to link the findings with health outcomes.

Fine particulate matter (PM_2.5_) is a major indicator of air pollution that poses the greatest risk to people’s health. Research shows that historical PM_2.5_ exposure is proportional to coronavirus disease 2019 (COVID-19) mortality rates.^1^ Most of the direct (ie, construction sites and fire) and indirect (ie, power plants, industries, and automobiles) sources of PM_2.5_ pollutants were shut down during the COVID-19 lockdown period. For this reason, a decline in PM_2.5_ pollution has been observed in nations responding to the COVID-19 pandemic.^2^

Bangladesh is the world’s most polluted country for PM_2.5_ exposure.^3^ Every year, several patients with cardiovascular and respiratory difficulties die due to unhealthy and hazardous PM_2.5_ pollution.^4^ Dhaka (the capital of Bangladesh) is the most vulnerable city to both PM_2.5_ pollution^5^ and COVID-19 transmission in Bangladesh.^6^ High population density combined with poor air quality makes the city risky for COVID-19 patients. Governments have taken different policies and initiatives regarding PM_2.5_ pollution control, but enormous construction projects and developments have hampered the government initiatives. As a result, the spending of government funds might have increased, but the PM_2.5_ pollution remains the same.

The first COVID-19-positive case was found in Dhaka on March 8, 2020, and like many other countries, the Bangladesh government declared a countywide lockdown from March 24, 2020. The lockdown resulted in not only reducing the rate of new COVID-19 cases but also in reducing the amount of air pollutants.^7^ But the identification of PM_2.5_ pollution change has not yet been clarified, so it is not obvious how much the PM_2.5_ pollution has decreased in Dhaka due to COVID-19 lockdown. In this situation, ground-based measurements and observation of PM_2.5_ pollutant concentrations might be an important tool for detecting and reducing pollution through regulatory compliance.

This research included investigating the PM_2.5_ scenarios and identifying changes during COVID-19 lockdown based on time series datasets. This study delineates the trend of PM_2.5_ along with the summary statistics for pre-COVID-19, during COVID-19, and post-COVID-19 lockdown periods for the study period (2017-2020). The research might help decision-makers to make effective policies to reduce the PM_2.5_ pollution based on the behavioral changes of the communities due to COVID-19. The specific objective of the research was to identify the impacts of COVID-19 on PM_2.5_ pollution change in the study area.

## Methods

Dhaka, the capital of Bangladesh, was selected as the study area. It is the ninth-largest and the sixth-most densely populated city in the world with a population of over 21 million.^8^ The city is known as the world’s second least livable city in terms of PM_2.5_ pollution. The research used hourly PM_2.5_ concentration of Dhaka from January 1, 2017, to August 1, 2020, which was collected from AirNOW data.^10^ The statistical software R was used for data preparation, visualization, and analysis. The collected hourly PM_2.5_ concentration data were converted to 24-h mean concentration. The trend of PM_2.5_ was investigated for the study period (2017-2020). The first 3 COVID-19-positive patients were confirmed in Bangladesh on March 8, 2020.^11^ The Bangladesh government declared a countrywide lockdown from March 24, 2020- and extended it to May 30, 2020. Based on the duration of the lockdown, the research classified the PM_2.5_ concentration data into pre-COVID-19 lockdown, during COVID-19 lockdown, and post-COVID-19 lockdown periods. For change detection, these 3 time periods were further sub-classified into 2 classes as current (data from 2020) and historical (data averaged from 2017 to 2019) data. Month-wise distribution of PM_2.5_ concentration for current and historical periods were investigated. Comparisons between historical versus current periods of PM_2.5_ concentrations were done using 2-sided t-tests. The research also reports both absolute differences and percentage change in pollution from historical to current periods.

## Results

The PM_2.5_ concentration showed a similar trend (ie, a sinusoidal pattern) for each of the years during the study period (2017-2020). The 24-h mean of PM_2.5_ was higher than the World Health Organization (WHO) standard (ie, 25 μg/m^3^) for the majority time in a year. Literature also shows that PM_2.5_ concentration is gradually increasing in Dhaka City.^12^ Rapid development, industrialization, and pollution were the major catalysts for the increase in PM_2.5_ concentration in the densely populated city.^13^ In recent years, a large number of mega projects, such as the metro rail, flyover, elevated expressway, etc., were undertaken in the city. Regular road construction and repair and other construction for fulfilling the faster-growing population needs, such as housing and utility services, have also increased in recent years. PM_2.5_ concentrations were higher in the start and the end of the year (ie, winter season), whereas relatively lower values were observed in the middle of the year (ie, rainy season). In the dry season (beginning and end of the year), the concentration of PM_2.5_ was higher than the other times of the year. Limited rainfall during this period allows pollutants to roam around in the air freely, which is 1 of the major reasons for higher PM_2.5_ concentration. Furthermore, development projects, such as the construction of road and drainage facilities, run in full swing at this time, which results in an increase in the concentration of PM_2.5_. On the contrary, the value was found low in the rainy season (ie, middle of the year) as raindrops clean the air. The maximum, average, and minimum values for PM_2.5_ (μg/m^3^) concentrations were found to be 324.96 μg/m^3^, 87.47 μg/m^3^, and 7.94 μg/m^3^, respectively, for the study period.

Table 1 illustrates the mean concentration of PM_2.5_ for both historical (2017-2019) and current (2020) period. In pre-COVID-19 lockdown period, historical mean of PM_2.5_ was 161.84 μg/m^3^, which has declined by 6.38% in current period compared with the historical average (*P* = 0.15; 95% confidence interval (CI): -24.36-3.4). The declining trend continued to the lockdown period when the mean concentration of PM_2.5_ declined by 11.31% (*P* = 0.10; 95% CI: -15.72-1.4). However, in post-COVID-19 lockdown period, the mean concentration stayed almost the same, and no statistically significant differences was found for this period (*P* = 0.94; 95% CI: -3.51-3.76).

**Table 1.** 24-h mean concentration of PM_2.5_ (μg/m^3^) in Dhaka City relative to COVID-19 lockdown periods

	Historical (2017-2019) Mean (SD)	Current (2020) Mean (SD)	Difference between current and historical means (% change)
Pre-COVID-19 lockdown (January 1 - March 23)	161.88 (58.12)	151.56 (54.14)	−10.32 (-6.38%)
During COVID-19 lockdown (March 24 - May 30)	63.23 (30.26)	56.07 (27.53)	−7.15 (-11.31%)
Post-COVID-19 lockdown (Mar 31 - August 1)	32.13 (13.23)	32.25 (10.87)	0.12 (0.40%)

**Figure 1.**
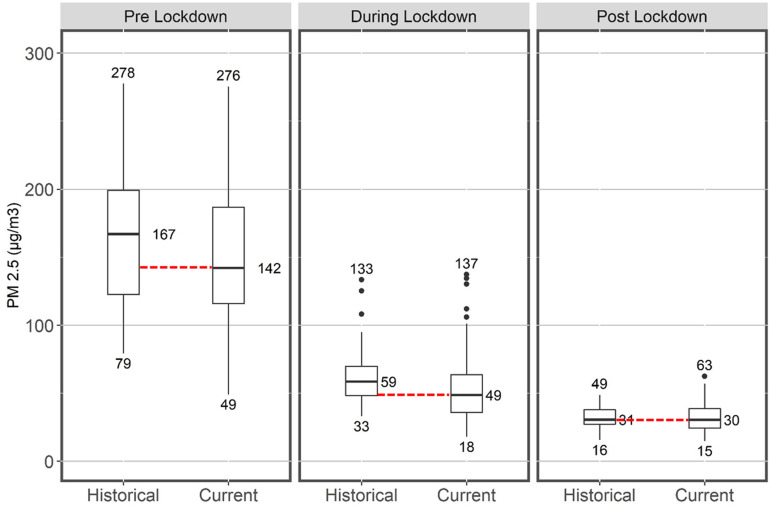
Summary statistics for 24-h mean values of PM_2.5_ (μg/m^3^) in current (2020) and historical (2017-2019) periods.

According to Figure 1, in the lockdown period, the median value of PM_2.5_ concentration declined at a higher rate than pre- and post-lockdown periods. During the lockdown, the median value of PM_2.5_ decreased by 17% in 2020 with respect to the historical median (2017-2019); the percentage change for the median was 15% and 3% in the pre- and post-lockdown period, respectively. If the median line is extended to the next chart within the group, it seems that there is unlikely to be a significant difference between historical data and current data as the extended median lines lie inside the interquartile range (IRQ) range for all 3 periods.

## Discussion

In the pre-COVID-19 lockdown period, the concentration of PM_2.5_ was higher at the start of the year. But the current period has a lower concentration than the historical period. It can be said that COVID-19 impact on PM_2.5_ started at the beginning of 2020. In this year, some of the mega projects were stopped or minimized due to a lack of foreign technology and manpower. The global impacts of COVID-19 also influenced industrial production. The majority of industries, such as garment industries, product orders were canceled during early 2020, as a result of which, the industrial source of PM_2.5_ might have been minimized. People were in fear of the pandemic; as a result, private vehicles on roads were limited during this period. In Dhaka, the decline of vehicles on roads might have affected the amount of PM_2.5_ concentration.

During the COVID-19 lockdown period, the amount of PM_2.5_ concentration declined in 2020 from the historical period. The government was forced to stop all offices, industries, and public transport, which might be the main cause of this decline. Electricity consumption was reduced during this period that might have an influence on the concentration of PM_2.5_. The literature also shows that the amount of PM_2.5_ in the air was reduced at a higher rate during COVID-19 lockdown period.^14^ The country’s economic structure was one of the major barriers to less amount of PM_2.5_ decline.^15^ During the middle and late lockdown period, daily earner people went to their work and small factories were restarted, violating the government restrictions. Due to the miscommunication between Bangladesh Garment Manufacturers and Exporters Association (BGMEA) and government bodies, some industries were reopened during the national lockdown. The industrial reopening might influence the amount of PM_2.5_ concentration in the city. Closing and reopening of industries, and major festivals, such as Eid, forced the local transport to start; as a result, the decline might be reduced in reference to the historical period. Some emergency repairing of roads and other utility facilities was done before the rainy season. Most of the mega projects were in an unfinished condition during this period. These issues might have an impact on the PM_2.5_ of the city.

In the post-COVID-19 lockdown period, all of the industries, office and construction work were reopened as in previous years. In this period, the concentration of PM_2.5_ was higher than the historical period. The unfinished construction work and pending orders in industries might have influenced the amount the PM_2.5_ in this period.

## Conclusions

COVID-19 lockdown has a significant impact on PM_2.5_ concentration as well as overall air quality. The mean PM_2.5_ concentration declined by 11.31% in current period compared with historical average. Community behavioral change and shut-down of PM_2.5_ pollutant sources played a vital role in reducing PM_2.5_ concentration during the COVID-19 lockdown. The research provides an overview of how the COVID-19 pandemic affected air pollution. The results will provide initial evidence regarding human behavioral changes and emission controls. The pandemic has already affected people’s lives in many ways. Researchers’ interest around the world regarding the pandemic and its associated impacts is increasing continuously. This research creates potential avenues for further study to link the findings with health outcomes. However, there are some limitations to this research. First, the research used only 3 y of data for calculating the historical trend. More historical data can provide a more accurate historical trend. Second, there could be other catalysts that can influence the PM_2.5_ concentration, which was not considered in this research. Future research should address these issues.
